# Supplementary material for: Inhibition increases response variability and reduces stimulus discrimination in random networks of cortical neurons
Source: Sci Rep. 2019 Mar 21;9:4969. doi: 10.1038/s41598-019-41220-2 (PMC6428807; doi:10.1038/s41598-019-41220-2)
Supplement: Supplementary file 1 — LaTeX Supplementary File [file 41598_2019_41220_MOESM1_ESM.pdf]

Supplementary Material for: 'Inhibition in Random Neuronal Networks Enhances Response Variability and Disrupts Stimulus Discrimination'

Netta Haroush[1,2] and Shimon Marom [1,2]

[1] Network Biology Research Laboratory, Electrical Engineering, Technion- Israel Institute of Technology, Haifa 32000, Israel.
[2]Department of Physiology, Biophysics and Systems Biology, Technion - Israel Institute of Technology, Haifa 32000, Israel.

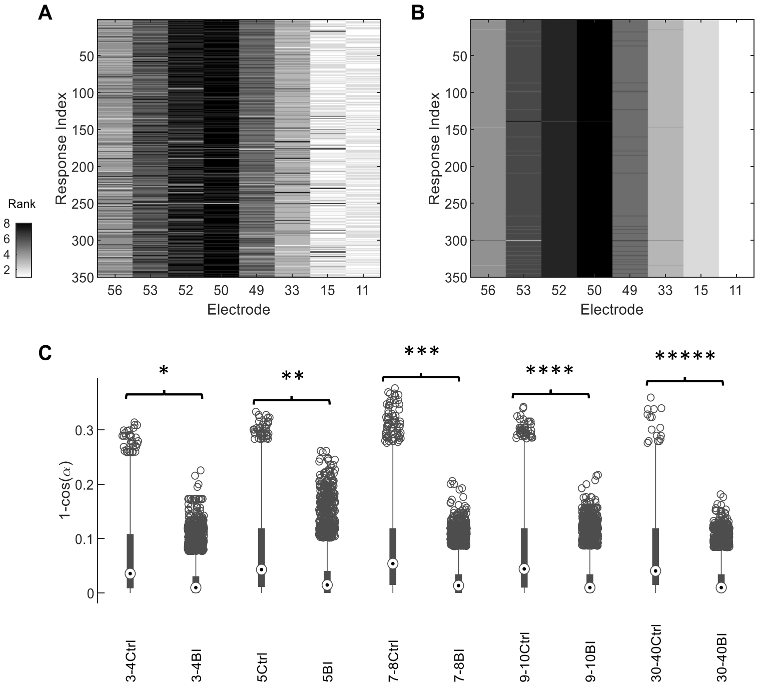

Figure S1: **Blocking inhibition reduces variability for any inter-stimulus-interval tested.** A: an instance from a single network of recruitment order vectors under control conditions at stimulation rate of 0.025-0.5hz. 50 order vectors are presented for every stimulation rate, such that responses to the fastest stimulation rate are shown at the bottom of the panel and responses under the slowest stimulation rate are at the top. B: the same after partially blocking inhibition. This is a special case where the network remained responsive also under very fast response rate (0.5hz, the bottom 50 lines). Since different networks varied in their responsiveness under different stimulation rates we pooled together responses from 4 networks at 5 bins of stimulation rate that were responsive for all networks, as indicated by the horizontal labels in Panel C. Stars indicate the identity of p-values indicating significant decrease in the distance distribution after blocking inhibition, using a right tail paired Wilcoxon signed rank test: p*=p**=p***=p****= p*****=0 (p is a 64 bits variable).

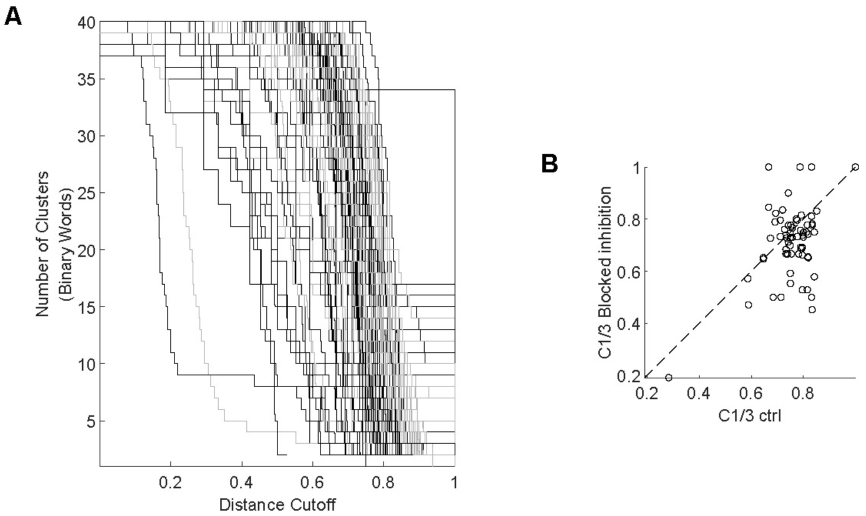

Figure S2: **Reduced variability of response patterns persist after correcting a possible sampling bias .** A: the number of clusters to which binary words are grouped, as a function of the distance cutoff used. Depicted in gray are data under intact inhibition whereas the black curves are displaying data under blocked inhibition, corrected for the increased gain (see text for further details). After correcting for the increased gain, the cluster content curves still show an overall left-shift in the presence of Bicuculline, indicating increased similarity between responses. B : the left shift is quantified for pairs of data from the same electrode under both conditions using C1/3 (see Fig3 caption), and indicates a decrease in variability for sub-sampling of binary words under blocked inhibition (p=0.0015 right tail of paired signed rank test).

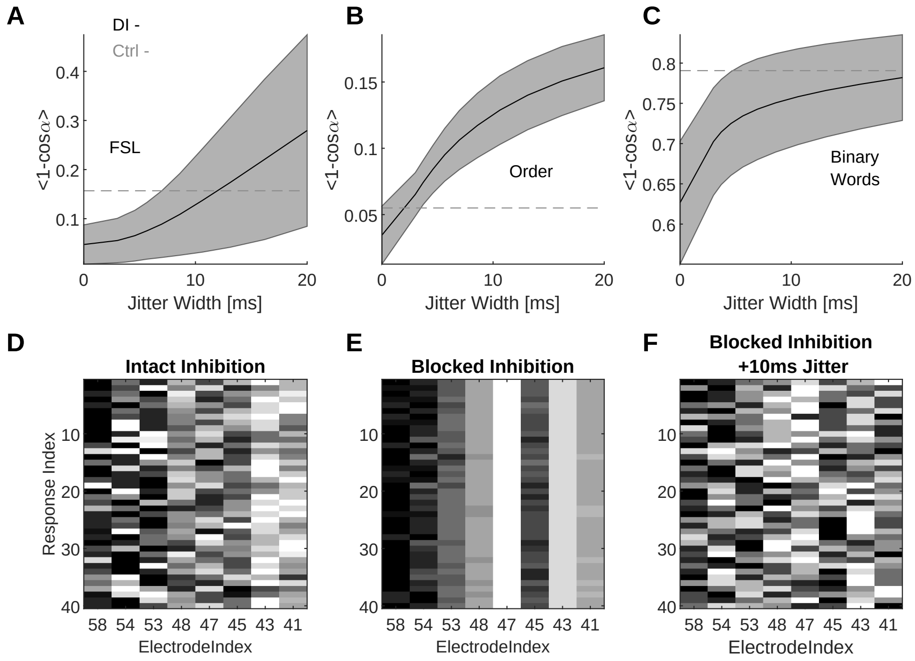

Figure S3: **The contribution of inhibition is estimated as equivalent to a 10ms jitter in spike time.** A: The mean distance $(1 - cos(\alpha)$, (α) is the angle between 2 response vectors) between FSL vectors as a function of the jitter added to spike trains recorded under blocked inhibition (n=8 sources in 8 networks). The black solid line depicts the mean distance as a function of the jitter; gray shades depict the standard deviation over the mean distances from all networks; the dashed line depicts the mean distance over all 8 networks under intact inhibition. B: the same for Order.C: the same for binary words. An order of ca.10ms jitter is bringing the mean distance between pairs of responses under blocked inhibition for all response features. D-F: an instance from a single network for recruitment order on repeated trials under control conditions (D), blocked inhibition (E) and under blocked inhibition with 10ms jitter (F). The simulated data in Panel F display comparable variability to that displayed by intact inhibition in Panel D.
