# Supplementary material for: Inhibition increases response variability and reduces stimulus discrimination in random networks of cortical neurons
Source: Sci Rep. 2019 Mar 21;9:4969. doi: 10.1038/s41598-019-41220-2 (PMC6428807; doi:10.1038/s41598-019-41220-2)
Supplement: Supplementary file 2 — Supplementary material [file 41598_2019_41220_MOESM2_ESM.pdf]

Inhibition increases response variability and reduces stimulus discrimination in random networks of cortical neurons

Netta Haroush[1,2,*] **and Shimon Marom**[1,2]

[1]Network Biology Research Laboratory, Electrical Engineering, Technion – Israel Institute of Technology, Haifa 32000, Israel
[2]Department of Physiology, Biophysics and Systems Biology, Technion – Israel Institute of Technology, Haifa 32000, Israel.
[*]nharoush@princeton.edu

ABSTRACT

Much of what is known about the contribution of inhibition to stimulus discrimination is due to extensively studied sensory systems, which are highly structured neural circuits. The effect of inhibition on stimulus representation in less structured networks is not as clear. Here we exercise a biosynthetic approach in order to study the impacts of inhibition on stimulus representation in non-specialized network anatomy. Combining pharmacological manipulation, multisite electrical stimulation and recording from *ex-vivo* randomly rewired networks of cortical neurons, we quantified the effects of inhibition on response variability and stimulus discrimination at the population and single unit levels. We find that blocking inhibition quenches variability of responses evoked by repeated stimuli and enhances discrimination between stimuli that invade the network from different spatial loci. Enhanced stimulus discrimination is reserved for representation schemes that are based on temporal relation between spikes emitted in groups of neurons. Our data indicate that – under intact inhibition – the response to a given stimulus is a noisy version of the response evoked in the absence of inhibition. Spatial analysis suggests that the dispersion effect of inhibition is due to disruption of an otherwise coherent, wave-like propagation of activity.

Introduction

Inhibition is a key determinant of structural and functional pattern formation in a wide range of biological phenomena[1–4]. In neural systems, inhibition seems to enrich the repertoire of activity patterns in the developing as well as the mature brain[5–10]; there is evidence to that effect also at the behavioral level[11–13]. Much of what is known of the impacts of inhibition in these systems comes from analyses of the sensory envelope. There, where the connectivity of inhibitory neurons is relatively stereotypic and where stimulus evoked activity can be meticulously analyzed, inhibition is underlying a core trait shared by all modalities: sharpening of stimulus selectivity and contrast sensitivity by means of lateral inhibition, which depends on a unique structural configuration[14–18]. Downstream the sensory envelope, where activity travels through less specialized structures, the impacts of inhibition on sensory processing are less clear, and the involvement of lateral inhibition is debated[19–22].

Here we investigate the impacts of inhibition on stimulus evoked activity, disentangled from the effects enforced by specialized structures. To this aim we record and analyze responses to stimuli before and after partial blocking of inhibition in large-scale random networks of cultured cortical neurons, using an array of extracellular electrodes. While 'not-a-brain', the setup offers an opportunity to study the effects of inhibition under weaker structural constrains, at high temporal resolution, in multiple sites and with good pharmacological control over the extent of inhibitory activity. Acknowledging the existence of many different mechanisms that potentially contribute to network inhibition[23,24], the present study implements control of inhibition by application of Bicuculline (blocking $GABA_A$ receptors and Ca^{2+} dependent K^+ channels). We studied the impacts of this pharmacological manipulation of inhibition on trial-to-trial variability and on discrimination between stimuli that invade the network from different spatial loci in the electrode array.

The reported results suggest that blocking inhibition in random networks reduces response variability and sharpens the sensitivity of activity to stimulus location. The extent to which inhibition impacts on stimulus discrimination depends on the type of response features taken into consideration. Interestingly, the response patterns exposed in the presence of Bicuculline share information with those detected in intact networks, suggesting that in a randomly rewired network inhibition acts as a disperser. The data indicate that the dispersion effect of inhibition is due to interference with wave-like propagation of activity through nearest neighbors, which is akin – in a certain sense – to the mechanism by which lateral inhibition sharpens stimulus contrast.

Results

Two related aspects of network response were studied: variability in responses to repeated stimuli delivered through the same spatial location (electrode, or 'site'), and separability of responses to stimuli delivered at different spatially located electrodes. In the latter (separability) set of experiments, we insisted on analyzing responses of the same group of neurons to the different stimulation sites, thus mimicking a case where different stimuli are processed by the same cell assembly.

Figure 1B shows a typical response to a single stimulus, detected through 60 electrodes. In order to quantify response variability and separability of inputs, two classes of response features were extracted: *rate* and *temporal relations* between spikes recorded in different electrodes. The rate features used are *population firing rate* summed over all electrodes (Figure 1A), and "*binary words*"[25] (Figure 1E), which are firing rate vectors picked by single electrodes binned such that every time window maximally contains a single action potential (thus bordering precise timing). As representatives of the class of temporal relations between spikes recorded at different electrodes, we used *first spike latencies* (FSL, Figure 1C) extracted from delays to first spike recorded in 8 of the most active electrodes; and *recruitment order* (Figure 1D), a subset of the FSL, specifying only the participation rank of each electrode of the FSL vector. For visual simplicity, only four of the most active electrodes (highlighted red in Figure 1B) were used in panels C–D. These compressed representations of different aspects of the raw data are used throughout the manuscript. See the Methods section for details on response features extraction.

Inhibition increases response variability

Responses to repeated input from a single stimulation source were recorded before and after bath application of 4–5μM Bicuculline (total of 8 sources from 8 different networks), a concentration that significantly reduces neuronal inhibition, but not to an extent leading to seizure-like activity[7,26] (see Methods). Trial-to-trial variability was evaluated under each condition: the intact and the inhibition-blocked network. As previously reported[27], in the presence of Bicuculline the networks show enhanced responsiveness to stimulation. Each response terminates within $\sim$1 second (and often less), congruent with the timescales of *in-vivo* cortical responses under anesthetized conditions where inhibition is reduced[28]. Blocking inhibition reduces variability across trials when compared to the intact network; this effect is observed in all response features tested. Figure 2 demonstrates quenched variability of both binary words (2A and B) and recruitment order (2C and D) features. As may be expected, FSL is impacted in a manner similar to recruitment order (not shown), and the reduced variability in population rate (not shown) agrees with previously reported observations in this preparation[27,29].

The reduction in response variability across our data set may be quantified by cluster analysis (see Methods): for each response feature and under each condition, a hierarchical tree of responses is constructed based on pairwise distances (illustrated in Figure 3A). We then systematically changed the cutoff distance, that is – the distance below which two responses are considered similar. Each dendrogram was then characterized by the rate at which the number of clusters decreases as a function of the cutoff distance (see examples of distance cutoffs in Figure 3A). The intuition being that in the one extreme, each observation (i.e. response feature vector) forms a completely isolated cluster on its own, whereas at the other extreme, all responses are identical and only one cluster exists. Reduction of response variability would then be reflected in a more rapid decline of number of clusters as cutoff distance is enlarged. This is the picture seen in Figure 3 (B and D), where blockade of inhibition causes an overall left shift of the cluster content curves, compared to control conditions. The effect is shown for both binary words (Figure 3B, n=64 electrodes in 8 networks) and recruitment order (Figure 3D, n=8 networks). The effect is also observed at the level of individual data sets, where pairs of control and blocked curves are compared (Figure 3C and 3E, respectively).

The data of Figures 2 and 3 were generated by stimulating the networks at a rate of 0.2 per second, but we documented the above reported effects at different stimulation rates in the same network (3–40 seconds intervals, $n = 4$ networks); indeed, within this frequency range variability is reduced for all stimulation rates and all features (Supplementary Fig. S1 is demonstrating this for recruitment order).

Blocking the inhibition in a neural network enhances neuronal response gain [20,30,31]. Thus, the stereotypic response patterns might be a trivial outcome of sampling bias: increased spiking probability that entails increased effective sampling rate of the "complete" spike time series. To evaluate the contribution of such sampling bias to our observations we sub-sampled the dataset, randomly erasing spikes from the time series recorded in each electrode in the Bicuculline blocked inhibition. The extent of subsampling was tuned to fix the average number of spikes for each electrode equivalent to that under control conditions. While this artificial sub-sampling does cause recovery of some variability in the Bicuculline blocked inhibition data, all the effects reported above persists (See supplementary Fig. S2).

Inhibition decreases stimulus discrimination

Given the stereotypic response to a single stimulation source, induced by blocking inhibition, how does blocking inhibition affects the discrimination of stimuli delivered from different spatial sources? One may imagine two extreme cases: (1) block of inhibition increases network symmetry, collapsing the response down to a single pattern, irrespective of the stimulation

site. Alternatively, (2) block of inhibition breaks network symmetry, generating a unique response to each input source; a stiff "lookup table". We thus proceeded to examine the network response to randomly alternating stimulation sites (n=9 networks, 30 different stimulation sites) before and after bath application of Bicuculline (2–8μM), and evaluated separability of responses to the different sources using unsupervised separation as well as supervised classification procedures. We were particularly interested in the more challenging task of stimulus discrimination that is based on the activity of non-selective neurons that respond to *all* stimulation sites. We therefor selected the group of electrodes in which responses were reliably detected for all stimuli, across conditions (see Methods), a context that may be mapped to a scenario of different stimuli being processed by the same cell assembly. As shown below, in the presence of Bicuculline discrimination based on the relation between spike times recorded in different electrodes becomes sharper; discrimination remained unchanged (on average) when based on rate features.

Unsupervised separation. The effect of inhibition on stimulus discrimination was quantified using the contrast between responses pooled from pairs of stimulation sources in an unsupervised protocol. The contrast measure (adopted from Beggs and Plenz, 2004) is calculated based on intra-source and inter-source distances (see Methods). Overall, the contrast between pairs of stimulation sources increases in the presence of Bicuculline when estimated from recruitment order (Figure 4A) and FSL (not shown), but not when estimated from population rate data (Figure 4B) or binary words of single electrodes (not shown).

Supervised classification. Support vector machine (SVM; see Methods) was implemented in order to evaluate discrimination of individual responses to stimuli delivered from two or four different sites. Congruent with the results of the above unsupervised protocol, classification accuracy is improved in the presence of Bicuculline when estimated from recruitment order (Figure 4C) and FSL (not shown), but not when estimated from population rate data (Figure 4D) or binary words of single electrodes (not shown). This result holds when implementing the SVM procedure on responses to two stimulation sites (black markers in Figure 4C and 4D) as well as four stimulation sites (red markers in Figure 4C and 4D). Note the several cases of deteriorated discrimination, when based on recruitment order (FSL) in the presence of Bicuculline; this point is revisited in the third part of the Result section.

The overall picture seems consistent with a scenario in which blocking the inhibition partitions the network to different, stiff propagation paths; hence discrimination that is based on spike time relations (but not rate) becomes sharper.

Inhibition as network noise generator

Trial to trial variability is often interpreted as reflecting 'machine noise'[32,33]. We examined the above-reported observations as reflecting noise contributed by inhibition to an otherwise (relatively) noise-less neural activity. To that end, a noisy version of the data recorded in the presence of Bicuculline was generated, by randomly jittering the spike-time series to varying widths. Indeed, a jitter of ca. $\sim$10 ms recovers the characteristic trial to trial response variability of the intact network (See supplementary Fig. S3); this is true for all response features tested. Viewed from that angle, blocking inhibition with Bicuculline exposes the "reproducible" version of a response, to which inhibition contributes noise. Congruent with this inhibition-as-network-noise-generator interpretation, SVM separation hyperplanes constructed from control discrimination experiments, succeeds surprisingly well to classify responses of the corresponding inhibition-blocked networks, and *vice versa*. This is demonstrated in cases where reducing the inhibitory activity had a significant impact on discrimination: FSL and recruitment order representation schemes (Figure 5A and B). These results suggest that spike time relations under both conditions share information. Together, these results strongly support the interpretation of network responses under intact inhibition being a "noisy" (or at least variable) version of reliable responses obtained in the absence of inhibition.

Further insight into ways by which inhibition contributes to response variability may be gained by looking at the spatial arrangement of network responses to stimuli in the presence of Bicuculline. High dosage of inhibition blockers induce wave-like activity[7,26,34]; a related phenomenon is observed under physiological conditions, during development, where inhibition has not yet assumed its re-polarizing impact[35]. Such coherent traveling waves, by themselves, would reduce trial to trial variability. Figure 6A depicts time to first spike following stimulation at the upper-right corner (colored red) of an intact (Bicuculline-free) network. Each square within the 6x10 array represents one electrode; the distance between the electrodes is $500\mu m$. Time to first spike is depicted by grayscale: black for short delay, white for a long delay. Note that in 6A, the electrodes in the immediate vicinity of the stimulation site are not the first ones to respond. The situation is different when Bicuculline is added (Figure 6B), where activity seems to propagate smoothly and directly to the nearest neighbors of the stimulation site. To quantify the effect across our dataset, we calculated the conditional firing probability (CFP): the probability of electrode j to detect a spike at a time interval τ following a spike detected in electrode i[36,37]. For each pair of recording electrodes, time to the peak of the CFP distribution (see left inset of Figure 6C) is taken to represent the characteristic delay between the spike times of detected in the two electrodes (see CFP in Methods). While the averaged delay increases with the physical distance for both conditions (data pooled from n=8 networks), there is a clear increase in delay between distal electrodes in the presence of Bicuculline (middle panel of Figure 6C). This impact is also apparent when examined for each individual network under the two conditions (right inset of Figure 6C).

It was previously shown that separability of input sources becomes sharper the more physically distant the stimulation sites

are[38]. Here we report that this correlation nearly doubles for recruitment order upon blocking the inhibition (Fig 6D). Note that in cases of wave-like propagation, separating responses to nearby stimulation sites might in fact deteriorate, as activity can propagate through similar (or overlapping) paths. Figure 6E suggests that cases where discrimination is not improved, or even deteriorates after blocking inhibition, are confined to shorter distances (ca. $\leqslant$ 2mm) between stimulation sources. These results suggest that the increased length scale of synchronization induced by blocking inhibition, sharpens the differences between responses to more distal inputs, while the resolution for distinguishing two adjacent inputs apart may deteriorate.

Discussion

We investigated the impacts of inhibition on the network capacity to reliably represent different stimuli, in the absence of specialized structure. To this aim, random networks of cortical neurons were stimulated at multiple loci, and the impacts of blocking inhibition – on both response variability and stimulus discrimination – were quantified. We show that blocking the inhibition causes quenching of response variability from any given source and for all response features tested. The reduction of variability accords with previous reports for single neurons under blocked inhibition, for both evoked and spontaneous activity[18,39]. At the network level, it has been recently demonstrated that blocking inhibition in this preparation induces highly reproducible population rate-profiles for both spontaneous and evoked activity[26,27,29]. Furthermore, this macroscopic effect is also reflected in a large body of work on neural avalanches, where blocking inhibition transforms heavy-tailed distributions of event size to narrow bimodal ones[40–43]. Our analyses demonstrate that the reduction in variability is also manifested at the microscopic level, in the fine spatio-temporal patterns of neural activity.

Under blocked inhibition discrimination that is based on the relations between spike times of different cells (i.e. FSL, recruitment order) is sharpened as a function of the distance between stimulation sites. Stimulus discrimination that is based on rate features is unaffected. Both of these observations are incongruent with the notion of inhibition as a means to enhance network ability to differentiate between stimuli. This discrepancy in the role taken by inhibition may be related to the target of our analyses: we were interested in how the *same* group of neurons processes different stimuli, and therefor focused on the less studied case of non-selective cells rather than the more extensively studied case of selective cells [21,44–46].

The overall reduced response variability and improved stimulus discrimination under blocked inhibition are accompanied by coherent traveling waves originating from the stimulation site. Traveling waves of comparable propagation velocity ($\sim$0.5m/s, Figure 6B) are abundant in sensory cortices [47–49], however their role is unknown and their existence is considered baffling in the light of stimulus selectivity of cortical cells. Our results suggest that traveling waves may engage a complementary representation strategy to that of stimulus selectivity — carrying information about the stimulus identity within the spatio-temporal activity patterns of *non-selective* cells.

The data is consistent with the interpretation that inhibition in large random networks mimics lateral inhibition, at least in the sense of interrupting the propagation of excitation to nearby neighbors. The spontaneous emergence of such effective lateral-inhibition impact in randomly rewired networks could arise from the fast and coherent manner by which inhibitory activity propagates through strong electrical coupling[50,51]. The spatial correlations involved are thus controlled by the extent of inhibition [28,52,53], a trace of which is seen in our analyses of CFP after blocking inhibition (albeit intermingled in our data with the dimensions of the electrode array used).

We further show that responses under control conditions are “noisy" (or variable) versions of the reproducible responses in the absence of inhibition. This conclusion is consistent with indications in the literature, pointing to inhibition as contributing to variability. Thus, for instance, inhibitory synapses change more rapidly[54], inhibitory neurons decorrelate network models[55,56], and blocking inhibition reduces variability[12,18,39].

Overall, the observations reported in this research imply that in random networks inhibition enhances the repertoire of activity patterns, shortens activation path between distal cells, and impacts on the resolution of telling stimuli apart. It is tempting to contemplate E/I ratio as a dynamic variable that may enhance or restrict network plasticity and enables adaptation of network states to tasks carried by the same cell-assembly[27,57–59].

Methods

Cell preparation.

Cortical neurons were obtained from newborn rats (Sprague-Dawley) within 24 hours after birth using mechanical and enzymatic procedures described in earlier studies[60]. Rats were anesthetized by CO_2 inhalation according to protocols approved by the Technion's ethics committee. All procedures involving cell preparation and animals handling were performed in accordance with these guidelines and regulations. The neurons were isolated and plated directly onto substrate-integrated multi electrode arrays. They were allowed to develop into functionally and structurally mature networks over a period of 2 weeks and were used in experiments within the period of 2–6 weeks post plating. The plated neurons cover an area of about 380 mm^2, bathed in Minimum Essential Medium Eagle (Sigma), supplemented with heat-inactivated horse serum (5%), glutamine (0.5 mM),

glucose (20 mM), and gentamycin (10 μg/ml), and maintained in an atmosphere of 37°C, 5% CO_2 and 95% air in an incubator as well as during the recording phases. An array of 60 Ti/Au extracellular electrodes, 30 μm in diameter, spaced 500 μm from each other (MultiChannelSystems, Reutlingen, Germany) was used. The insulation layer (silicon nitride) is pretreated with polyethyleneimine (Sigma, 0.01% in 0.1 M Borate buffer solution).

Electrophysiology & Pharmacology.

A commercial amplifier (MEA-1060-inv-BC, MCS, Reutlingen, Germany) with frequency limits of 150–3,000 Hz and a gain of x1024 was used for obtaining data. Data was digitized using an acquisition board (PD2-MF-64-3M/12H, UEI, Walpole, MA, USA). Each channel, sampled at a frequency of 16 kHz, detects electrical activity that might be originated from several sources (typically 1–3 neurons) as the recording electrodes were surrounded by several cell bodies. Action potentials originating from cell bodies are typically an order of magnitude stronger than axonal action potentials[61], we used threshold-based algorithm for spike detection, therefor our recording conditions are strongly biased to picking up signals from cell bodies. We have used a Simulink-based software for on-line control of data collection [see Zrenner et al. (2010) for details]. Voltage stimulation was applied in the form of a mono-phasic square pulse 200 μsec 800–950 mV through extracellular electrodes using a dedicated stimulus generator (MCS, Reutlingen, Germany). Action potentials timestamps were detected on-line by threshold crossing of negative voltage. Detection of synchronous events (Network Spikes, NSs) was performed off-line using a previously described algorithm[62] based on threshold crossing of the network firing rate (binned to 3 msec). Once a NS was detected within 400 millisecond following a stimulus, action potentials recorded in all the electrodes within 500 ms following the stimulus were extracted. Post-stimulus time histograms were constructed using a 1 ms time bin, and smoothed with a 5 ms moving average; responses were then screened for a maximum amplitude of at least 1.5 spikes/ms. As indicated above, the recording conditions were kept identical to the incubation conditions; i.e: bathed in Minimum Essential Medium Eagle (Sigma) that was supplemented with heat-inactivated horse serum (5%), glutamine (0.5 mM), glucose (20 mM), and gentamycin (10 μg/ml), and maintained in an atmosphere of 37°C, 5% CO_2 and 95% air. We performed two types of experiments: in the first, repeating stimuli were applied through a single electrode at a constant interval (5-8 seconds), under control conditions (intact inhibition) and in the presence of Bicuculline, a blocker of fast GABA transmission (4-5μM, n=8 networks). In the second type of experiments we provided stimuli from several electrodes (2, 3 or 4 different electrodes; altogether 9 networks) in random orders but at constant intervals (4-8 seconds), under control conditions and blocked inhibition (2-8μM Bicuculline). In 2 out of 9 networks used for stimulus classification experiments, the basal responsiveness was low; in these two cases we have used 1μM Bicuculline to increase baseline responsiveness. For blocked inhibition conditions, Bicuculline bath application was administrated as following: we first added 1 or 2μM of Bicuculline, and waited 10 minutes to make sure that the substance impact has stabilized, and then shortly tested the responsiveness from all sources. We then decided whether to add more Bicuculline based on 3 indicators for the level of the blockade: response probability, response latency and response duration[27]. In case response latency was still longer then ca. 50ms, and response probability has not increased for all sources, we increased the dose in 2μM steps. In all our experiment, responses did not sustain beyond ca. one second.

Data Analysis.

Action potentials recorded within 500 ms following each stimulus were used for the extraction of different response features. Generally, a minimum participation in 90% of stimuli was set as a limit for inclusion of a channel (electrode) in the analysis of all response features; in analyses of population rate, all electrodes were used. *First spike latencies* were calculated from a subset of the 8 most active electrodes. In cases of a failure to participate in a response, a random value was assigned, pooled from a uniform distribution over the interval [10-500] ms. This choice was taken in order to avoid trivialization of response classification by *support vector machine* (SVM) according to the missing electrodes. Similar results of the agglomerative analysis (see below) are obtained when these random values were replaced with zeros.

Recruitment order was defined as the rank of the electrodes, ordered according to their first spike latencies; cases of missed response were assigned an averaged rank (for illustration see Fig1B). *Binary words* were extracted for single electrodes from the 250 ms post stimulus using a 2 ms resolution, resulting in binary vectors. *Population rate* was calculated as described above over the range of 10-500 ms post stimulus (1 ms resolution, smoothed with 5 ms moving average).

Distances were computed for pairs of response feature vectors with a $1 - cos(\alpha)$ metric. Similar results to those reported here were obtained using other metrics (Levenshtein, correlation, or euclidean). Distances were computed for 40 responses from each of the single source experiments (n=8 networks), and 40 responses from each source for experiments with multiple stimulation sources (n=9 networks).

Supervised and unsupervised classification procedures were used to quantify dispersion of responses from single sources, as well as for evaluating separability of responses from multiple stimulation sources. To evaluate response dispersion for repeated inputs from a single source, a hierarchic tree was constructed for each network (agglomerative clustering procedure, with $1 - cos(\alpha)$ metric); on each iteration the most proximal distance value was taken as the distance between pairs of clusters. Cutoffs limiting the maximal distance allowed between two clusters were implemented. To evaluate separability of responses

from multiple sources, a *Contrast* measure adopted from Beggs and Plenz (2004) was calculated for pairs of sources (n=40 per source, 30 sources in 9 networks, overall 38 pairs of sources), defined as follows:

$Contrast = \frac{D_{out}-D_{in}}{D_{out}+D_{in}}$

where D_{in} is the sum of distances between all pairs of responses to a given stimulation source, D_{out} being the sum of distances between all pairs of inter-source responses (responding to two different stimulation sources), using the distance metric of $1-cos(\alpha)$. Note that this measure relies on the ground truth of stimulation identity, and thus only evaluates the relative goodness of separation across conditions.

Support Vector Machine (SVM) with a Gaussian radial basis function (RBF) kernel, as well as a linear kernel, were used to classify responses from 2 or 4 sources (n=40 responses per source). Classification performances were averaged over 50 repetitions for each classification process (50% validation set). For SVM of population rate histograms with a Gaussian radial-based kernel, only the 10-70 ms range were used.

Conditional Firing Probabilities (CFP)[36,37] were computed for all pairs of electrodes, with a minimal activity threshold of 100 spikes per electrode (within 100 ms from stimulus onset). Binary representations of single electrode firing rate were generated with a temporal resolution of 2 ms. The conditioned probability for electrode i to fire within a delay of $t=\tau$, given that electrode j fired at t=0, was computed for $\tau=[0,100]$ at 2 ms steps. The resulting profiles of the CFP were then smoothed with a moving average of 3 consecutive values. The first maximum of each profile was extracted along with its index, which is referred to as the "typical delay". CFP profiles with a maximum lower then 0.05 were excluded. The typical delays were then averaged for distances between electrode i and j over data pooled from 8 networks.

Statistical inference was used to support the trends we recognize as being induced by blocking inhibition. In all cases, at least one of the datasets (i.e control or blocked inhibition) stray from a normal distribution at the tails. We therefore opted to use only non parametric tests. We used *paired Wilcoxon signed rank test* for either right or left tail, as indicated in the text where the distribution evaluated is *[control feature] -[blocked inhibition feature]*. Wherever correlation between 2 variables is estimated, we used *Spearman correlation*. P-values and correlation values are stated within figure captions.

References

1. Oster, G. F. Lateral inhibition models of developmental processes. *Math. Biosci.* **90**, 265 – 286, DOI: https://doi.org/10.1016/0025-5564(88)90070-3 (1988).
2. Cross, M. C. & Hohenberg, P. C. Pattern formation outside of equilibrium. *Rev. modern physics* **65**, 851 (1993).
3. Ball, P. & Borley, N. R. *The self-made tapestry: pattern formation in nature*, vol. 198 (Oxford University Press Oxford, 1999).
4. Meinhardt, H. & Gierer, A. Pattern formation by local self-activation and lateral inhibition. *Bioessays* **22**, 753–760 (2000).
5. Ben-Ari, Y. Excitatory actions of gaba during development: the nature of the nurture. *Nat. Rev. Neurosci.* **3**, 728–739 (2002).
6. Feller, M. B. Spontaneous correlated activity in developing neural circuits. *Neuron* **22**, 653–656 (1999).
7. Steriade, M. & Contreras, D. Spike-wave complexes and fast components of cortically generated seizures. i. role of neocortex and thalamus. *J. neurophysiology* **80**, 1439–1455 (1998).
8. Chervin, R., Pierce, P. & Connors, B. Periodicity and directionality in the propagation of epileptiform discharges across neocortex. *J. Neurophysiol.* **60**, 1695–1713 (1988).
9. Buzsáki, G. & Wang, X.-J. Mechanisms of gamma oscillations. *Annu. review neuroscience* **35**, 203 (2012).
10. Shew, W. L., Yang, H., Yu, S., Roy, R. & Plenz, D. Information capacity and transmission are maximized in balanced cortical networks with neuronal avalanches. *J Neurosci* **31**, 55–63, DOI: 10.1523/JNEUROSCI.4637-10.2011 (2011).
11. Hikosaka, O. & Wurtz, R. H. Modification of saccadic eye movements by gaba-related substances. i. effect of muscimol and bicuculline in monkey superior colliculus. *J. Neurophysiol.* **53**, 266–291 (1985).
12. Matsumura, M., Sawaguchi, T., Oishi, T., Ueki, K. & Kubota, K. Behavioral deficits induced by local injection of bicuculline and muscimol into the primate motor and premotor cortex. *J. neurophysiology* **65**, 1542–1553 (1991).
13. Jackson, G. M., Draper, A., Dyke, K., Pépés, S. E. & Jackson, S. R. Inhibition, disinhibition, and the control of action in tourette syndrome. *Trends cognitive sciences* **19**, 655–665 (2015).
14. Hartline, H. K., Wagner, H. G. & Ratliff, F. Inhibition in the eye of limulus. *The J. general physiology* **39**, 651–673 (1956).
15. Barlow, H. & Levick, W. R. The mechanism of directionally selective units in rabbit's retina. *The J. physiology* **178**, 477–504 (1965).

16. Olsen, S. R. & Wilson, R. I. Lateral presynaptic inhibition mediates gain control in an olfactory circuit. *Nature* **452**, 956–960 (2008).

17. Arevian, A. C., Kapoor, V. & Urban, N. N. Activity-dependent gating of lateral inhibition in the mouse olfactory bulb. *Nat. neuroscience* **11**, 80–87 (2008).

18. Najac, M. *et al.* Intraglomerular lateral inhibition promotes spike timing variability in principal neurons of the olfactory bulb. *The J. Neurosci.* **35**, 4319–4331 (2015).

19. Poo, C. & Isaacson, J. S. population code of odors in rat olfactory cortex, also suggest global inhibition instead of lateral inhibition for contrast enhancemnet. *Neuron* **62**, 850–861 (2009).

20. Isaacson, J. S. & Scanziani, M. How inhibition shapes cortical activity. *Neuron* **72**, 231–243 (2011).

21. Katzner, S., Busse, L. & Carandini, M. Gabaa inhibition controls response gain in visual cortex. *The J. neuroscience* **31**, 5931–5941 (2011).

22. Tremblay, R., Lee, S. & Rudy, B. Gabaergic interneurons in the neocortex: From cellular properties to circuits. *Neuron* **91**, 260 – 292, DOI: https://doi.org/10.1016/j.neuron.2016.06.033 (2016).

23. O'Leary, T., Williams, A. H., Franci, A. & Marder, E. Cell types, network homeostasis, and pathological compensation from a biologically plausible ion channel expression model. *Neuron* **82**, 809–821 (2014).

24. Marder, E., O'Leary, T. & Shruti, S. Neuromodulation of circuits with variable parameters: Small circuits reveal principles of state-dependent and robust neuromodulation. *Annu. Rev. Neurosci.* **37**, 329–346 (2014).

25. Schneidman, E., Berry, M. J., II, R. S. & Bialek, W. Weak pairwise correlations imply strongly correlated network states in a neural population. *Nature* **440**, 1007 (2006).

26. Keren, H. & Marom, S. Long-range synchrony and emergence of neural reentry. *Sci. reports* **6**, 36837 (2016).

27. Haroush, N. & Marom, S. Slow dynamics in features of synchronized neural network responses. *Front. computational neuroscience* **9** (2015).

28. Haider, B., Häusser, M. & Carandini, M. Inhibition dominates sensory responses in the awake cortex. *Nature* **493**, 97–100 (2013).

29. Baltz, T. & Voigt, T. Interaction of electrically evoked activity with intrinsic dynamics of cultured cortical networks with and without functional fast gabaergic synaptic transmission. *Front. cellular neuroscience* **9** (2015).

30. Wilson, N. R., Runyan, C. A., Wang, F. L. & Sur, M. Division and subtraction by distinct cortical inhibitory networks in vivo. *Nature* **488**, 343 (2012).

31. Olsen, S. R., Bortone, D. S., Adesnik, H. & Scanziani, M. Gain control by layer six in cortical circuits of vision. *Nature* **483**, 47 (2012).

32. Faisal, A. A., Selen, L. P. & Wolpert, D. M. Noise in the nervous system. *Nat. Rev. Neurosci.* **9**, 292–303 (2008).

33. McDonnell, M. D. & Ward, L. M. The benefits of noise in neural systems: bridging theory and experiment. *Nat. Rev. Neurosci.* **12**, 415–426 (2011).

34. Huang, X. *et al.* Spiral wave dynamics in neocortex. *Neuron* **68**, 978–990 (2010).

35. Blankenship, A. G. & Feller, M. B. Mechanisms underlying spontaneous patterned activity in developing neural circuits. *Nat. Rev. Neurosci.* **11**, 18–29 (2010).

36. Eytan, D., Minerbi, A., Ziv, N. & Marom, S. Dopamine-induced dispersion of correlations between action potentials in networks of cortical neurons. *J. neurophysiology* **92**, 1817–1824 (2004).

37. le Feber, J. *et al.* Conditional firing probabilities in cultured neuronal networks: a stable underlying structure in widely varying spontaneous activity patterns. *J Neural Eng* **4**, 54–67, DOI: 10.1088/1741-2560/4/2/006 (2007).

38. Kermany, E. *et al.* Tradeoffs and constraints on neural representation in networks of cortical neurons. *J Neurosci* **30**, 9588–9596, DOI: 10.1523/JNEUROSCI.0661-10.2010 (2010).

39. Häusser, M. & Clark, B. A. Tonic synaptic inhibition modulates neuronal output pattern and spatiotemporal synaptic integration. *Neuron* **19**, 665–678 (1997).

40. Beggs, J. M. & Plenz, D. Neuronal avalanches in neocortical circuits. *J Neurosci* **23**, 11167–11177 (2003).

41. Mazzoni, A. *et al.* On the dynamics of the spontaneous activity in neuronal networks. *PloS one* **2**, e439 (2007).

42. Shew, W. L., Yang, H., Petermann, T., Roy, R. & Plenz, D. Neuronal avalanches imply maximum dynamic range in cortical networks at criticality. *J Neurosci* **29**, 15595–15600 (2009).

43. Gautam, S. H., Hoang, T. T., McClanahan, K., Grady, S. K. & Shew, W. L. Maximizing sensory dynamic range by tuning the cortical state to criticality. *PLoS Comput. Biol* **11**, e1004576 (2015).

44. Sillito, A. The contribution of inhibitory mechanisms to the receptive field properties of neurones in the striate cortex of the cat. *The J. physiology* **250**, 305–329 (1975).

45. Nelson, S., Toth, L., Sheth, B. & Sur, M. Orientation selectivity of cortical neurons during intracellular blockade of inhibition. *Science* **265**, 774–777 (1994).

46. Li, G., Yang, Y., Liang, Z., Xia, J. & Zhou, Y. Gaba-mediated inhibition correlates with orientation selectivity in primary visual cortex of cat. *Neuroscience* **155**, 914–922 (2008).

47. Sato, T. K., Nauhaus, I. & Carandini, M. Traveling waves in visual cortex. *Neuron* **75**, 218–229 (2012).

48. Reimer, A., Hubka, P., Engel, A. K. & Kral, A. Fast propagating waves within the rodent auditory cortex. *Cereb. Cortex* **21**, 166–177 (2010).

49. Muller, L., Chavane, F., Reynolds, J. & Sejnowski, T. J. Cortical travelling waves: mechanisms and computational principles. *Nat. Rev. Neurosci.* (2018).

50. Gibson, J. R., Beierlein, M. & Connors, B. W. Functional properties of electrical synapses between inhibitory interneurons of neocortical layer 4. *J. neurophysiology* **93**, 467–480 (2005).

51. Mancilla, J. G., Lewis, T. J., Pinto, D. J., Rinzel, J. & Connors, B. W. Synchronization of electrically coupled pairs of inhibitory interneurons in neocortex. *The J. Neurosci.* **27**, 2058–2073 (2007).

52. Petersen, R. S., Panzeri, S. & Diamond, M. E. Population coding of stimulus location in rat somatosensory cortex. *Neuron* **32**, 503–14 (2001). 0896-6273 (Print) Journal Article Research Support, Non-U.S. Gov't.

53. Karnani, M. M. *et al.* Opening holes in the blanket of inhibition: localized lateral disinhibition by vip interneurons. *J. Neurosci.* **36**, 3471–3480 (2016).

54. Villa, K. L. *et al.* Inhibitory synapses are repeatedly assembled and removed at persistent sites in vivo. *Neuron* **89**, 756–769 (2016).

55. King, P. D., Zylberberg, J. & DeWeese, M. R. Inhibitory interneurons decorrelate excitatory cells to drive sparse code formation in a spiking model of v1. *J. Neurosci.* **33**, 5475–5485 (2013).

56. Tetzlaff, T., Helias, M., Einevoll, G. T. & Diesmann, M. Decorrelation of neural-network activity by inhibitory feedback. *PLoS computational biology* **8**, e1002596 (2012).

57. Fu, Y. *et al.* A cortical circuit for gain control by behavioral state. *Cell* **156**, 1139–1152 (2014).

58. Letzkus, J. J., Wolff, S. B. & Lüthi, A. Disinhibition, a circuit mechanism for associative learning and memory. *Neuron* **88**, 264–276 (2015).

59. Jovanic, T. *et al.* Competitive disinhibition mediates behavioral choice and sequences in drosophila. *Cell* **167**, 858–870 (2016).

60. Marom, S. & Shahaf, G. Development, learning and memory in large random networks of cortical neurons: lessons beyond anatomy. *Q. reviews biophysics* **35**, 63–87 (2002).

61. Holt, G. R. & Koch, C. Electrical interactions via the extracellular potential near cell bodies. *J. computational neuroscience* **6**, 169–184 (1999).

62. Eytan, D. & Marom, S. Dynamics and effective topology underlying synchronization in networks of cortical neurons. *J Neurosci* **26**, 8465–8476 (2006).

Author contributions statement

N.H. and S.M. conceived the work, N.H. conducted the experiments, and N.H and S.M. analysed the results and wrote the manuscript.

Additional information

Competing interests

The authors declare no competing interests.

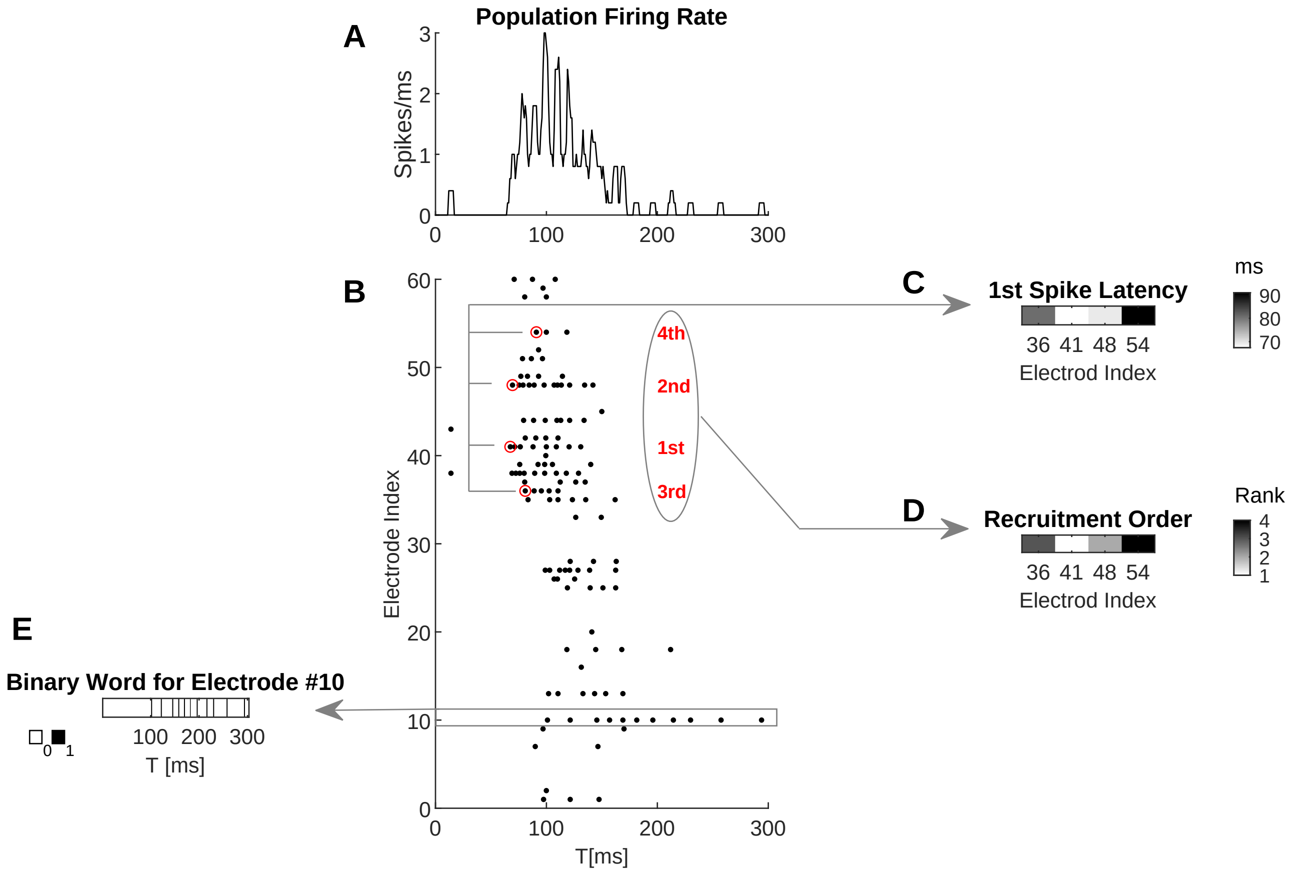

Figure 1. Response features and terminology. Network response to a single stimulus, recorded through 60 electrodes during 300 ms post stimulus; the stimulus is applied at $t = 0$. The extraction of response features, subjects to further analyzes in the manuscript, is schematically demonstrated in the different panels. A: **population rate** is summed over all electrodes shown in B, where each dot represents an action potential detected in one of the electrodes. C: **first spike latency** is extracted in this example from the first spikes detected in 4 electrodes (depicted by red circles). D: **recruitment order** is extracted by ranking the electrodes according to their order of first spike appearances (1st, 2nd, etc.). E: Spike time series from single electrodes are converted into **binary words**. Further details are available in the Method section.

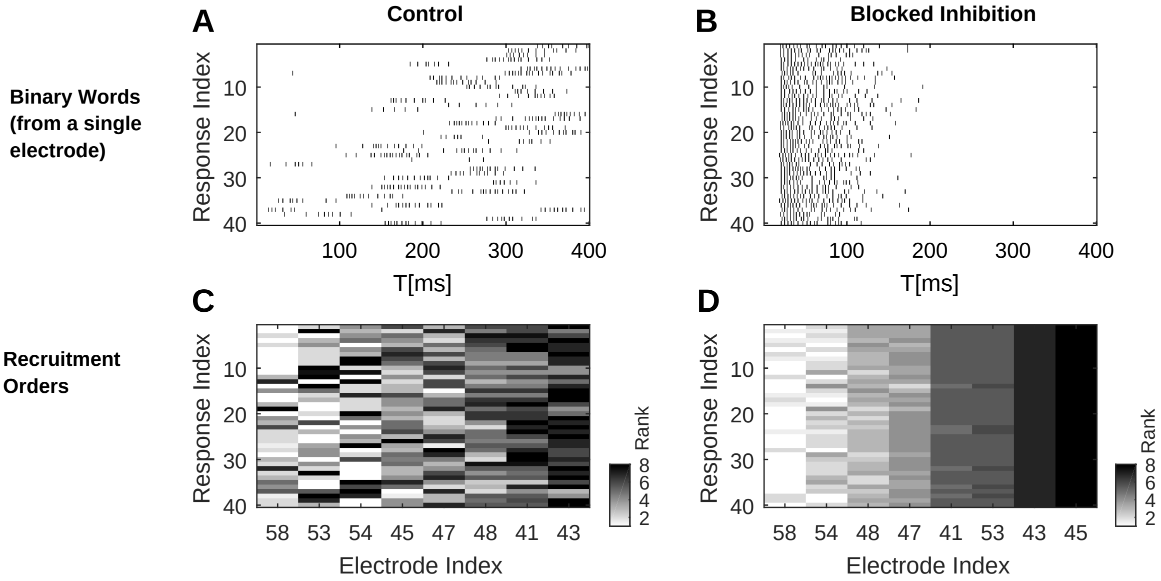

Figure 2. Blocking inhibition reduces trial-to-trial variability in response to repeated stimuli from a single stimulation site. A: a series of responses picked by a single electrode under control conditions, represented as **binary words** (each line depicts one trial). B: Responses of the same electrode in the presence of (4–5μM) Bicuculline. C: a series of **recruitment order** vectors for 8 of the most active electrodes in the same network, in response to the same stimulation series, under control conditions. Electrodes index is sorted according to their average rank of participation. D: **recruitment order** vectors of the same eight electrodes, in the presence of Bicuculline.

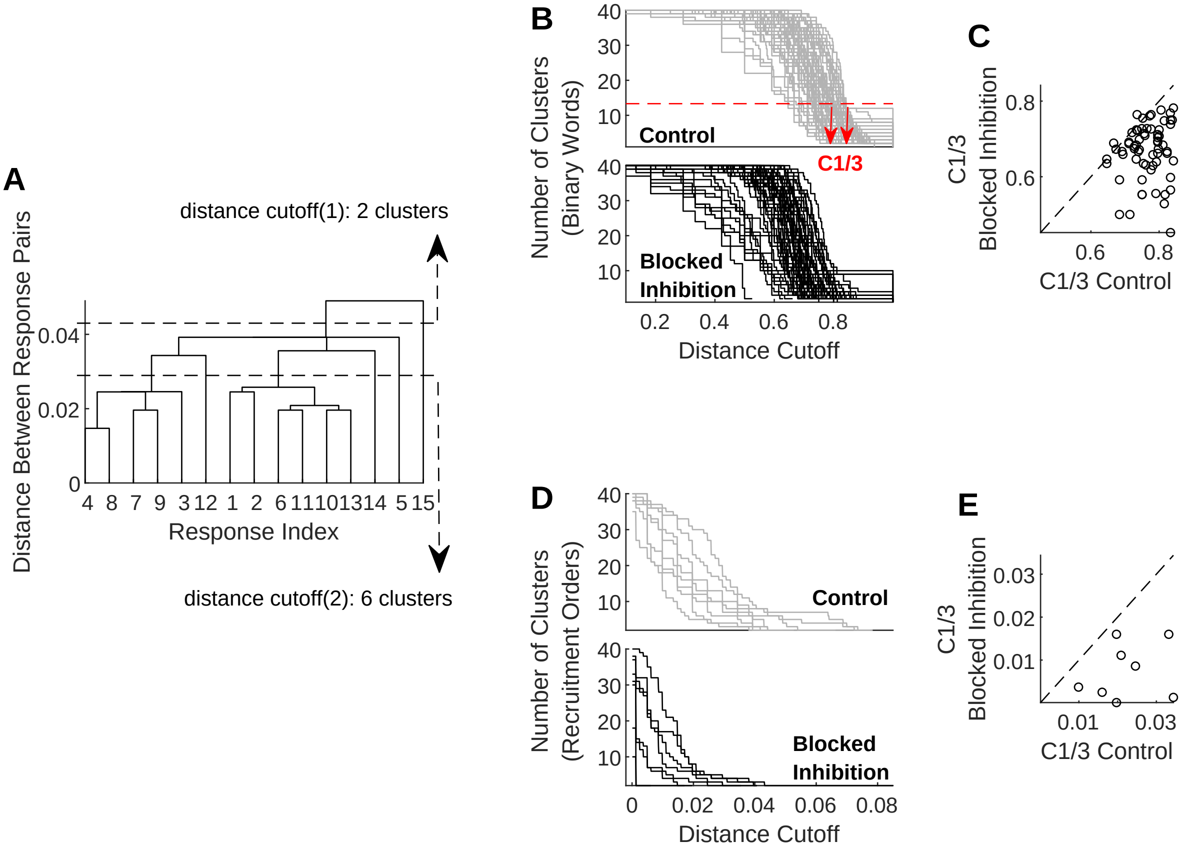

Figure 3. Bicuculline quenches the cluster content of activity patterns in response to a single stimulation site. Distances were computed for all possible pairs of responses in each network ($n = 8$ networks, 40 responses each), and a dendrogram tree was constructed (for binary words, responses from each electrode were used to construct a dendrogram, $n = 64$ electrodes). A: an instance of such a dendrogram. Cluster content was estimated by systematically changing the distance cutoff along the y-axis, and counting the resulting cluster number. Cutoff(1) and cutoff(2) illustrate the cluster counting procedure, resulting in two and six clusters, respectively. B: the number of clusters to which **binary words** are grouped, as a function of the distance cutoff used. The cluster content curves show an overall left-shift in the presence of Bicuculline, indicating increased similarity between responses. D: same for **recruitment orders**. C: quantifies the left shift of pairs of cluster-distance curves obtained before and after the blockade, for the case of binary words. $C_{1/3}$ is the cutoff value for which the number of clusters drops below one third of the maximal cluster number (illustrated in red arrows on Panel B). Under blocked inhibition, nearly all cluster-distance curves show a marked decrease of $C_{1/3}$ ($p = 9.5564^{-12}$, right tail of paired signed rank test, see Statistical inference in Methods), as expected for more similar responses. E: same for recruitment orders ($p = 0.0039$, right tail of paired signed rank test).

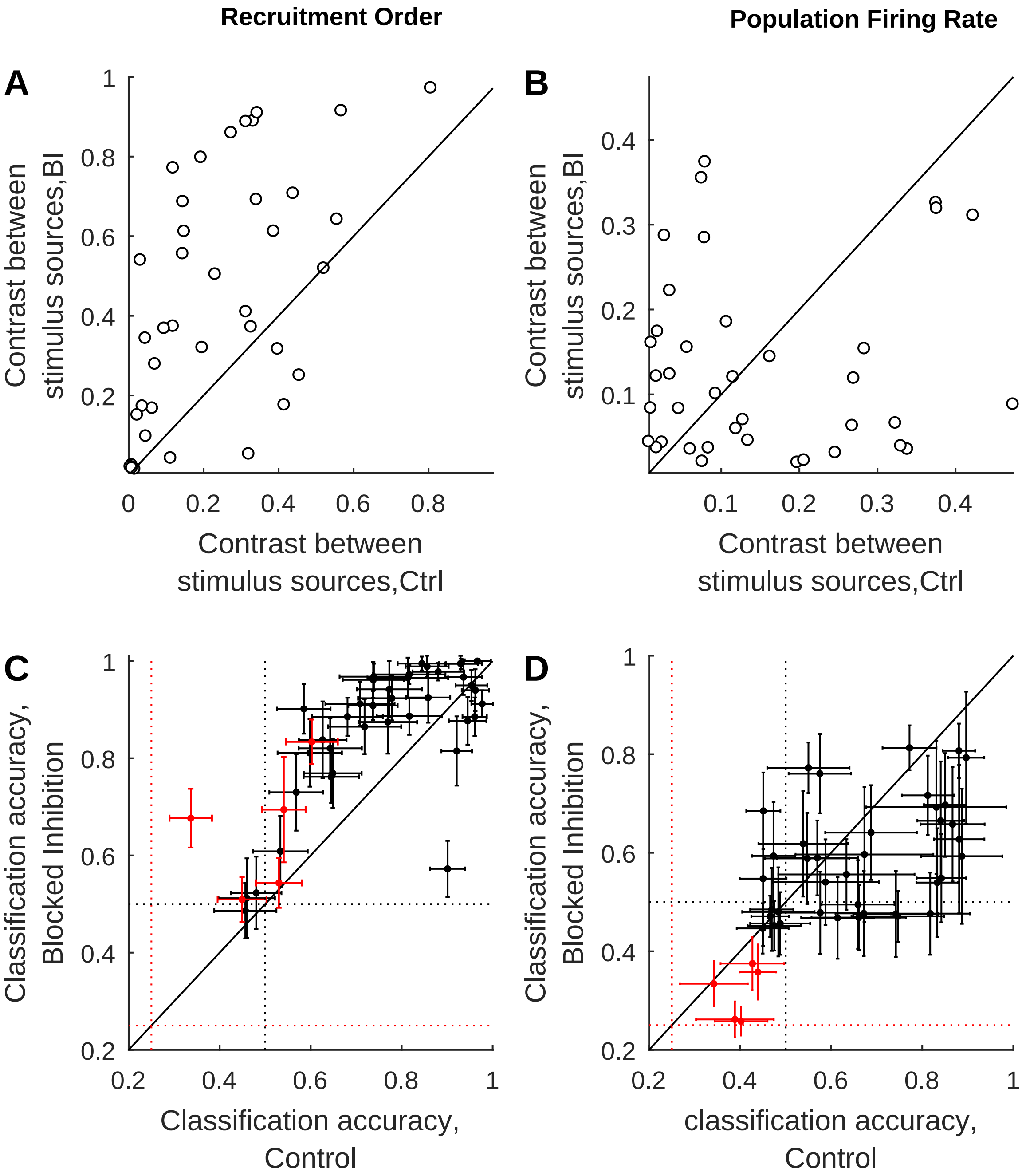

Figure 4. Blocking inhibition improves classification accuracy of stimulus location when based on recruitment orders, but not when based on population firing rates. Comparison of stimulus discrimination under blocked inhibition and control conditions. A&B: the contrast (see Methods) between pairs of stimulation sources (9 networks, $n = 38$ sources, 40 responses from each), on control (Ctrl) and blocked inhibition (BI) conditions. The contrast is enhanced upon blocking inhibition in the case of recruitment order (panel A, $p = 1.3016^{-5}$, left tail of paired signed rank test), but not in the case of population rate (panel B, $p = 0.5$, two tails paired signed rank). Similar results are obtained in supervised clustering (using SVM, see methods), as demonstrated in Panels C and D. Here, classification accuracy is presented for pairs of stimulation sources (black, $n = 38$ pairs from 9 networks) and for groups of 4 stimulation sources (red, $n = 5$ networks); error-bars indicate accuracy standard-deviation over repeated classification trials, for which training and test sets are randomized. Dotted lines depict chance levels for pairs of sources (black) and for groups of 4 sources (red). Solid black lines mark unity in all panels.

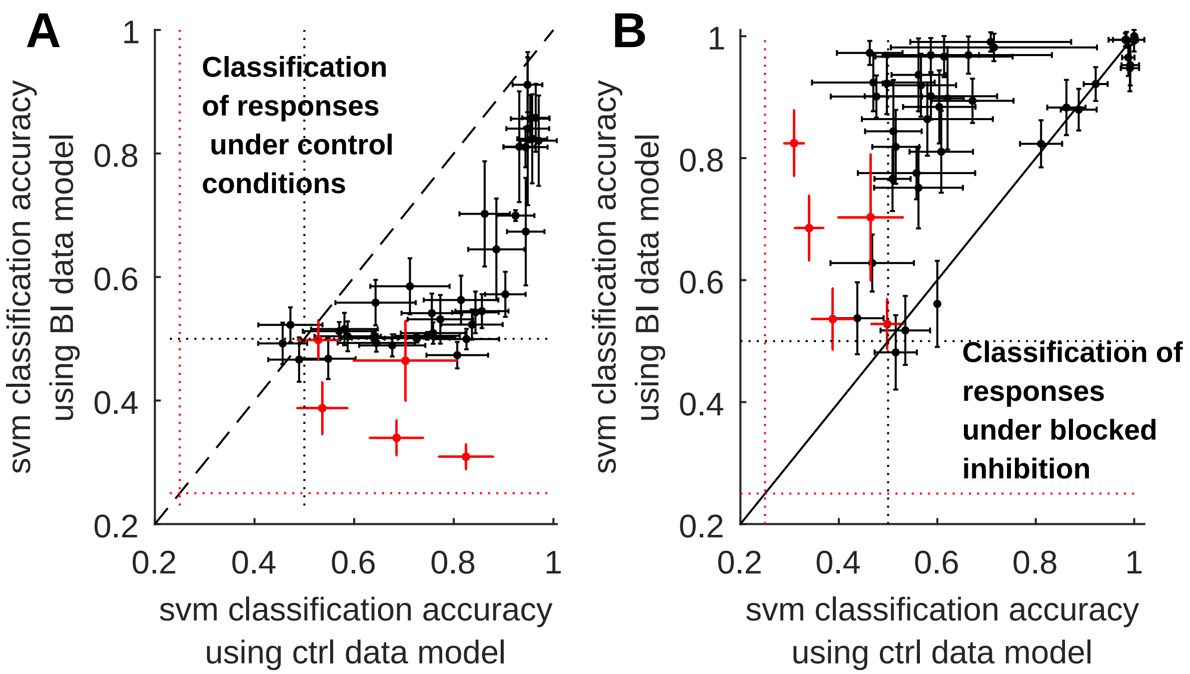

Figure 5. Response patterns in Bicuculline treated and intact networks are related to each other. A: SVM separation hyperplanes constructed from control discrimination experiments, classify responses of the corresponding inhibition-blocked networks, and *vice versa* (B). Chance levels are depicted by dotted lines. Black depicts $n = 38$ two-source discrimination, and red depicts four-sources discrimination ($n = 5$ networks). Note in panel A that the correlation between classification accuracies approaches unity in the easier (two-sources) discrimination task. Similar results are obtained for first spike latencies (not shown).

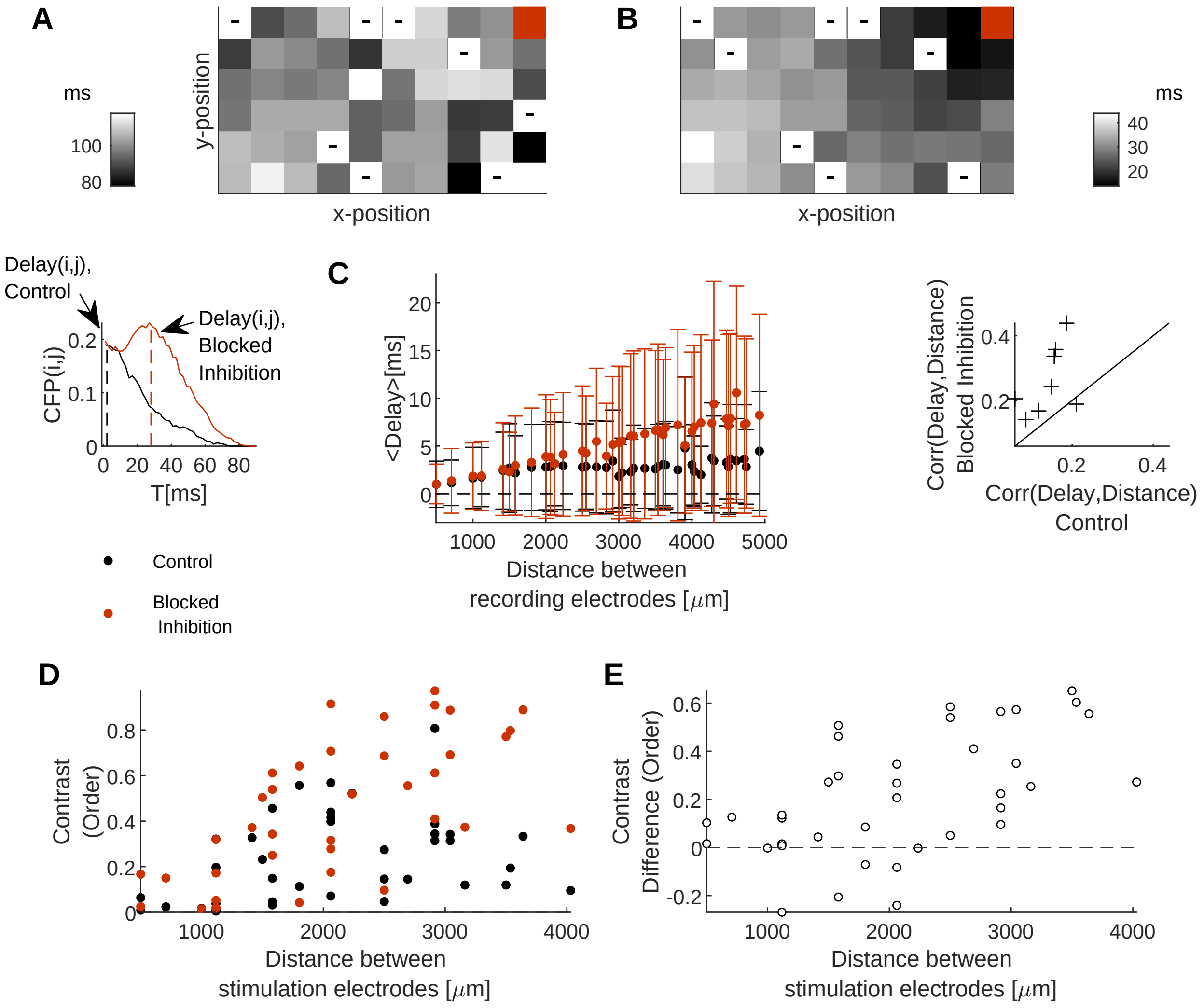

Figure 6. Bicuculline enhances propagation of activity through near neighbors. A: depicts time to first spike following stimulation at the upper-right corner (colored red) of an intact (Bicuculline-free) network. Each square within the 6x10 array represents one electrode; the distance between the electrodes is $500\mu m$. Time to first spike is depicted by grayscale (dark is fast to respond, clear is late to respond; see inset and note different grayscale for panel A and B); a minus sign indicates that no response was detected in that electrode. Note that in panel A, the electrodes in the immediate vicinity of the stimulation site are not the first ones to respond. The situation is different when Bicuculline is added (B), where activity seems to propagate smoothly and directly to the nearest neighbors of the stimulation site. C: Conditional firing probability (CFP), the probability of electrode j to detect a spike at a time interval τ following a spike detected in electrode i. For each pair of recording electrodes, time to the peak of the CFP distribution (see left inset of C) is taken to represent the characteristic delay between the spike times of detected in the two electrodes. The averaged delay increases with the physical distance for both conditions (data pooled from $n = 8$ networks), but there is a clear increase in delay between distal electrodes in the presence of Bicuculline (middle panel of C). Spearman correlation between the delay and the distance for individual networks, under blocked inhibition *vs.* control conditions (C, right inset, $p = 0.0078$ left tail of paired signed rank test). D: contrast between responses to pairs of stimulation sites, as a function of the distance between them, under control (black, Spearman correlation $= 0.41$, $p = 0.0052$) and blocked inhibition (red, Spearman correlation$= 0.7$, $p = 5.3775^{-7}$) conditions. Subtracting the control contrast from the blocked inhibition contrast (E) exposes that blocking inhibition may worsen discrimination for adjacent stimulation sources, suggesting that inhibition contributes to a finer spatial resolution for telling stimuli apart.
